# The effects of chest drainage on pressure-controlled ventilation

**DOI:** 10.1186/s40981-022-00568-7

**Published:** 2022-10-03

**Authors:** Yuko Matsumoto, Shinju Obara, Takahiro Hakozaki, Tsuyoshi Isosu, Satoki Inoue

**Affiliations:** Department of Anesthesiology and Division of Intensive Care, Fukushima Medical University, 1 Hikarigaoka, Fukushima, Fukushima 960-1295 Japan

**Keywords:** Chest drainage, Pressure-controlled ventilation, Transpulmonary pressure

## Abstract

**Background:**

The use of pressure-controlled ventilation (PCV) for anesthesia management is becoming more commonly used. Chest drainage is commonly performed after thoracic surgery, and the negative pressure it generates might affect the transpulmonary pressure (TPP). In the present study, we investigated how chest drainage could affect ventilating conditions during PCV.

**Methods:**

We created a hand-made simple thoracic and lung model, which was connected to an anesthesia machine. The tidal volume (TV) was measured with positive end-expiratory pressure (PEEP) 0 and no chest drainage (baseline), followed by 10 cmH_2_O PEEP/no drainage, 10 cmH_2_O PEEP/drainage with − 10 cmH_2_O and 10 cmH_2_O PEEP/drainage with − 20 cmH_2_O. Finally, TV with 20 cmH_2_O and 30 cmH_2_O PEEP/no drainage was measured. Driving (inspiratory) pressure was maintained at 20 cmH_2_O during the whole experiment.

**Results:**

TV was significantly increased by applying 10 cmH_2_O PEEP compared with baseline, further increased by applying − 10 cmH_2_O by drainage, similar to the value with PEEP 20 cmH_2_O with no drainage (end-tidal TPP of 20 cmH_2_O for both). TV decreased to < 50% of the baseline by applying 10 cmH_2_O PEEP with − 20 cmH_2_O by drainage, which was similar to that with 30 cmH_2_O PEEP with no drainage (end-tidal TPP of 30 cmH_2_O for both).

**Conclusions:**

TV was maintained at similar levels with the same TPP, regardless of PEEP or negative pressure by chest drainage change, suggesting that negative intrapleural pressure by the chest tube drainage system might mimic PEEP from the point of TV.

## Introduction

Chest tube placement is a common procedure in the clinical setting to drain fluid, blood, or air from the pleural cavity. The negative pressure is usually applied to the drainage system, which generates negative pressure in the intrapleural space. Therefore, the negative pressure generated by the chest tube drainage system might affect the transpulmonary pressure (TPP), which is defined as the difference between alveolar and intrapleural pressure [1]. The formula is expressed as “TPP = alveolar pressure—intrapleural pressure.” Because the intrapleural negative pressure generated by the chest tube drainage system is a continuous pressure, it is supposed that it might work as positive end-expiratory pressure (PEEP). Therefore, the drainage of the lung would appear to be the result of a higher PEEP setting than the actual setting that was applied. Such situations could affect ventilation conditions. However, to the best of our knowledge, there have been no reports to date regarding this concern. Therefore, in the present study, we investigated how intrapleural negative pressure generated by the chest tube drainage system could affect ventilation conditions during pressure-controlled ventilation (PCV).

## Materials and methods

**Fig. 1:**
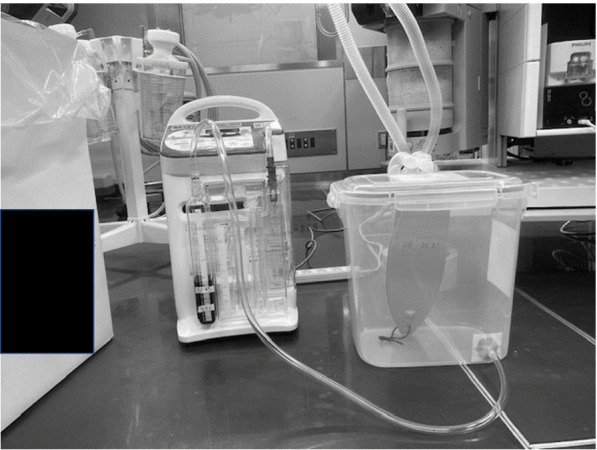
Experimental thoracic-lung model and the drainage system

To conduct this study, we created a simple thoracic and lung model. We used a commercially available sealed plastic container (22 cm × 33.3 cm × 30.5 cm) for storing and preserving food as a thoracic cage and a 1-l ventilator test lung (Venti.Plus™, GaleMed Corporation, Taiwan), whose compliance was 20 ml/cmH_2_O according to the manufacturer’s instructions, as an experimental lung. We made a hole on the cover of the container and attached a standard elbow connector, which had a 15-mm I.D./22-mm outer diameter (O.D), with strong glue gel so that we did not spoil the airtightness of the container. The test lung was connected through the elbow connector in the thoracic cage model and a disposable anesthesia breathing circuit was connected through the elbow connector on the top of the cage. In addition, we made a smaller hole on the side of the container and attached a 1.5-m flexible tubing with strong glue gel, which was connected to the chest tube drainage system (MERA continuous suction unit MS-009, Senko Medical Instrument Mfg. Co., Ltd. Tokyo). We used GE Datex Ohmeda Aestiva 5 (GE Healthcare Japan, Tokyo) as an anesthesia ventilator (Fig. 1).

### Experimental protocol

**Fig. 2:**
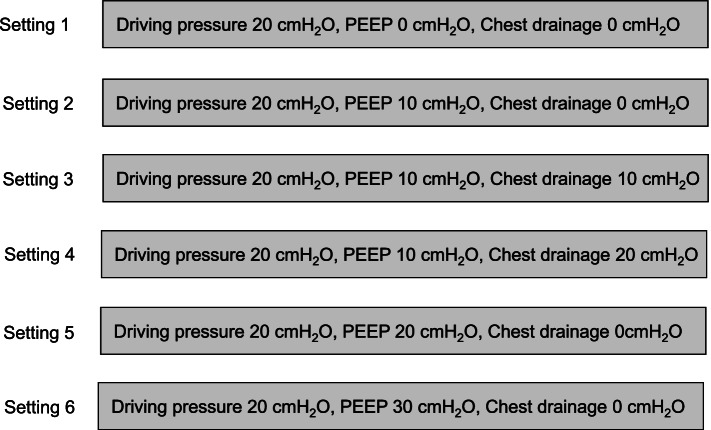
Experimental protocol. PEEP, positive end-expiratory pressure

Experimental PCV was started at the following setting; the driving pressure was set at 20 cmH_2_O with 0 cmH_2_O of PEEP. Then, tidal volumes (TVs) were measured sequentially 10 times by GE/Datex Ohmeda Flow Sensor (GE Healthcare Japan, Tokyo) mounted in Aestva 5, and their average was recorded. The respiratory rate (RR) 15 breaths/min and inspiratory-to-expiratory time ratio (I:E ratio) 1:2 were selected and were not changed during the study. Next, 10 cmH_2_O PEEP was applied to this ventilator setting, and the TVs were measured and averaged. In addition, chest drainage was applied at 10 and 20 cmH_2_O through the chest tube drainage system. Then, TVs were measured sequentially 10 times and the average was recorded. Finally, the chest drainage was ceased, PEEP was increased up to 20 and 30 cmH_2_O, and TVs were measured and averaged (Fig. 2). The theoretical peak-TPP (P-TPP) of each setting was calculated. Moreover, we defined end-expiratory TPP (EE-TPP) as the TPP at the end of expiration and calculated the theoretical EE-TPP at each ventilator setting. In this model, driving pressure with PEEP and drainage pressure were considered alveolar pressure and intrapleural pressure for convenience, respectively.

### Statistical analysis

All measured values are described as mean (standard deviation; SD). Comparisons between the groups were analyzed by one-way analysis of variance (ANOVA) followed by Bonferroni’s test. All statistical analyzes were performed with EZR (Saitama Medical Center, Jichi Medical University), which is based on R (The R Foundation for Statistical Computing) and R commander [2], and *P* values of < 0.05 were considered statistically significant. The sample size calculation was not done because this study was an observational exploratory research study.

## Results

**Fig. 3:**
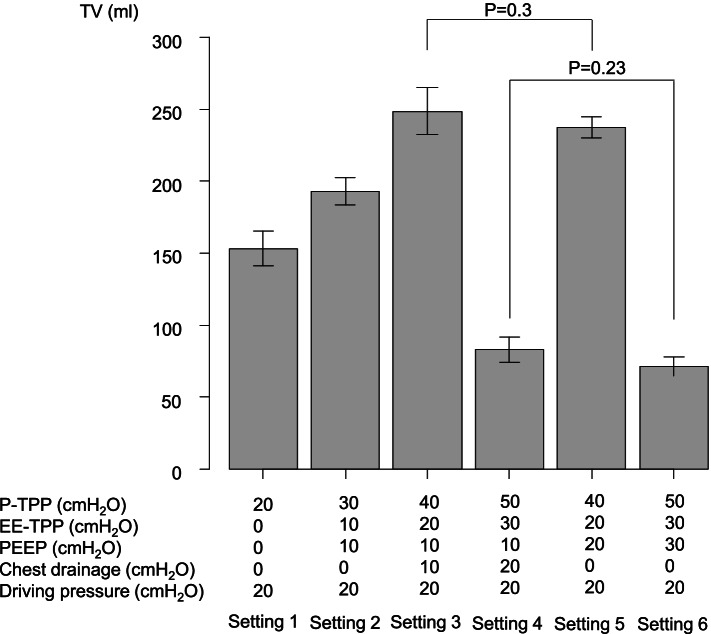
Experimental results. PEEP, positive end-expiratory pressure; P-TPP, peak transpulmonary pressure; EE-TPP, end-expiratory transpulmonary pressure

The theoretical P-TPPs of each setting (1–6) were 20 cmH_2_O, 30 cmH_2_O, 40 cmH_2_O, 50 cmH_2_O, 40 cmH_2_O, and 50 cmH_2_O, respectively. In addition, the theoretical EE-TPPs of each setting (1–6) were 0 cmH_2_O,10 cmH_2_O, 20 cmH_2_O, 30 cmH_2_O, 20 cmH_2_O, and 30 cmH_2_O, respectively (Fig. 3). According to our calculations, the theoretical EE-TPP at 10 cmH_2_O of PEEP combined with 10 cmH_2_O of the chest drainage was equivalent to that at 20 cmH_2_O of PEEP without chest drainage. In addition, the EE-TPP at 10 cmH_2_O of PEEP combined with 20 cmH_2_O of chest drainage was equivalent to that at 30 cmH_2_O of PEEP without chest drainage.

Compared to the basal setting, which was 20 cmH_2_O of driving pressure accompanied by 0 cmH_2_O of PEEP (setting 1), the TVs significantly increased after 10 cmH_2_O of PEEP was applied (setting 2). Additionally, the TVs further increased after the commencement of 10 cmH_2_O of chest drainage (setting 3); however, the TVs inversely decreased to less than half of the basal volume by applying an additional 10 cmH_2_O, which meant 20 cmH_2_O of chest drainage (setting 4). The TVs generated by 20 cmH_2_O of driving pressure accompanied by 20 cmH_2_O of PEEP (setting 5) were not statistically different from those generated by 20 cmH_2_O of driving pressure accompanied by 10 cmH_2_O of PEEP under 10 cmH_2_O of chest drainage (setting 3) (*P* = 0.3). The TVs significantly decreased after applying an additional 10 cmH_2_O, which meant 30 cmH_2_O of PEEP (setting 6); however, the TVs were not statistically different from those generated by 20 cmH_2_O of driving pressure accompanied by 10 cmH_2_O of PEEP under 20 cmH_2_O of chest drainage (setting 4) (*P* = 0.23). Although TVs generated by the ventilator settings between setting 3 versus 5 and setting 4 versus 6 were not different, any other pairwise comparisons of TVs generated by the ventilator settings showed significant differences (*p* < 0.0001, respectively).

## Discussion

Even using the same driving pressure during PCV, this experimental study revealed that TVs fluctuated when PEEP or chest drainage was applied. In addition, when applying any PEEP or chest drainage, this study showed that TVs did not significantly change when neither P-TPP nor EE-TPP was changed. Moreover, the TVs decreased even though the same driving pressure was used when EE-TPP was too high.

As mentioned before, TPP is defined as the difference between alveolar and intrapleural pressure [3]. Therefore, we presumed that the continuous negative pressure generated by the chest tube drainage system could affect TPP and might work like PEEP. Thus, when theoretical EE-TPPs, which were thought to work like PEEP, were not changed, it was confirmed that TVs were not changed if the same driving pressure was applied. On the other hand, in cases where theoretical EE-TPPs were changed, it was observed that TVs fluctuated even when the same driving pressure was used, where TVs increased in proportion to EE-TPP increasing, and finally decreased drastically with considerably high EE-TPP. This phenomenon is probably based on the characteristics of the pressure–volume (P–V) curve of the test lung used in the current study. (In the original concept of the P–V curve, “pressure” means “alveolar pressure”; however, it is better for “pressure” to be considered [4].) This means that the P–V curve of the test lung had a lower inflection point. It is known that lung compliance drastically changes when pressure passes the vicinity of a lower inflection point [3, 4]. This seems to explain why the TVs increased in proportion to EE-TPP in the current study. The reason why the TVs decreased drastically with considerably high EE-TPP even when the same driving pressure was applied may be because the test lung had an upper inflection point around 50 cmH_2_O of its own P–V curve. Otherwise, we might have missed this point between 30 and 40 cmH_2_O of pressure, since we did not measure TVs in this range. Because TPP above the upper inflection point shows disproportionate increases in TVs due to overdistension of the lung [4, 5], TVs decreased drastically even with the same driving pressure. It has become increasingly accepted that TPP is more useful than apparent PEEP and driving pressure when considering respiratory mechanics in mechanically ventilated lungs. However, in the clinical setting that continuous negative intrapleural pressure by chest drainage generates continuous positive TPP may be missed, which can move the locus on each patient’s own P–V curve. Especially, in cases with left-shifted P–V curves, overdistension of the lung may easily occur even under lower EE-TPP. Consequently, TVs may decrease after the commencement of chest drainage in such a situation. We have experience in managing a case of significant TV reduction after commencement of chest tube drainage under PCV following left lower lobectomy [6]. It was suspected that such a phenomenon occurred because continuous negative intrapleural pressure by chest drainage caused overextension of the lung, which increased elastance. Table 1 shows ventilating parameters in this case report [6]. In this case, TV decreased by 10 cmH_2_O of chest drainage even with the same driving pressure and PEEP because both P-TPP and EE-TPP were changed by the commencement of chest drainage. To gain the same TV, an increase in driving pressure, which meant an increase in P-TPP, was required because of increased elastance by overextension, which was generated by an increase in EE-TPP by the commencement of chest drainage. In fact, the conversion of PCV to VCV was done to gain the same TV. As a result, the driving pressure needed to increase twofold. This case report suggests that alteration of P-TPP and EE-TPP by chest drainage would generate different TVs even though the same driving pressure and PEEP are used in the clinical case as well as the mechanical model. Besides, it also suggested that different P-TPP would be required to maintain the same TV when EE-TPP is changed by chest drainage even though the same PEEP is used. We need to keep in mind that chest tube drainage might affect ventilation conditions by altering TPP.

**Table 1:** Ventilating parameter in the case report referred in the “Discussion” section [6]

	Before drainage	After drainage	Conversion of PCV to VCV
P-TPP (cmH_2_O)	14	24	34
EE-TPP (cmH_2_O)	4	14	14
PEEP (cmH_2_O)	4	4	4
Chest tube drainage (cmH_2_O)	0	10	10
Driving Pressure (cmH_2_O)	10	10	20
TV (ml)	450	250	450

*P-TPP* peak transpulmonary pressure, *EE-TPP* end-expiratory transpulmonary pressure, *PEEP* positive end-expiratory pressure, *TV* tidal volume, *PCV* pressure-controlled ventilation, *VCV* volume-controlled ventilation

In this case, TV decreased by 10 cmH_2_O of the chest drainage even with the same driving pressure and PEEP because both P-TPP and E-TPP were changed by the commencement of the chest drainage. To gain the same TV, an increase in driving pressure, which meant an increase in P-TPP, was required because of increased elastance by overextension, which was generated by an increase in E-TPP by the commencement of chest drainage

This was an experimental study using a hand-made thoracic-lung model. Therefore, it is not certain whether the continuous negative pressure generated by the chest tube drainage system could work in humans like this experimental model. In addition, the pressure range tested in this experiment is far greater than in the real clinical world. The reason why we used this pressure range is that extreme settings may be easier to understand the drastic changes of TV. However, we believe that our findings may be worthwhile reporting. Our study showed that TVs fluctuated by applying PEEP or chest drainage even when the same driving pressure is used during PCV. On the other hand, the TVs did not significantly change whenever neither P-TPP nor EE-TPP was changed when using the same driving pressure. Chest drainage is extremely familiar to many physicians and is frequently used in the clinical setting; however, it is imperative to understand whether continuous negative intrapleural pressure generated by the chest tube drainage system might mimic PEEP and affect respiratory mechanics in mechanically ventilated lungs.

## Data Availability

The datasets used and analyzed during the current study are available from the corresponding author on reasonable request.

## Abbreviations

TV: Tidal volume
TPP: Transpulmonary pressure
PEEP: Positive end-expiratory pressure
PCV: Pressure-controlled ventilation
P-TPP: Theoretical peak-TPP
EE-TPP: End-expiratory TPP
SD: Standard deviation
ANOVA: Analyzed by one-way analysis of variance
